# Calorie Restriction in Mammals and Simple Model Organisms

**DOI:** 10.1155/2014/308690

**Published:** 2014-05-06

**Authors:** Giusi Taormina, Mario G. Mirisola

**Affiliations:** Dipartimento di Biopatologia e Biotecnologie Mediche e Forensi (DiBiMeF), Università di Palermo, Via Divisi 83, 90133 Palermo, Italy

## Abstract

Calorie restriction (CR), which usually refers to a 20–40% reduction in calorie intake, can effectively prolong lifespan preventing most age-associated diseases in several species. However, recent data from both human and nonhumans point to the ratio of macronutrients rather than the caloric intake as a major regulator of both lifespan and health-span. In addition, specific components of the diet have recently been identified as regulators of some age-associated intracellular signaling pathways in simple model systems. The comprehension of the mechanisms underpinning these findings is crucial since it may increase the beneficial effects of calorie restriction making it accessible to a broader population as well.

## 1. Introduction

The amount and quality of nutrient intake during lifetime are commonly regarded as main health-span regulators. Diet is in fact one of the lifestyle components capable of affecting the quality and the duration of life in a wide range of living organisms. The list of human pathologies, directly or indirectly affected by nutrients, is growing at a fast pace and includes major causes of mortality and morbidity such as cardiovascular diseases, diabetes, cancer, inflammation and neurodegeneration. Considering that population aging and disabilities are major concerns industrialized countries are going to face in next years, the possibility to increase the health-span with a consequent reduction of related healthcare costs is of general interest. It is therefore surprising that the most straightforward nutritional intervention to prolong lifespan is almost 80 years old but has had only limited application so far.

McCay published, in 1935, the first paper demonstrating that reduced intake of nutrients without malnutrition (Calorie Restriction, CR) could increase the mean as well as the maximum lifespan of rats [1]. The amount of calorie deprivation and the age at which the reduction in calorie intake starts influence the magnitude of the modification observed. Many other investigators, throughout the world, have confirmed this observation in all the other model systems tested. Yeasts, fruit flies, nematodes, fishes, hamsters, and several strains of mice as well as rats consistently increase their lifespan when the nutrient availability drops between 30% and 75% of the normal calorie supplementation, according to the species considered. Not only calorie restricted rodents lived longer than the ad libitum fed counterparts, but a significant part of them (about 30%) died without any apparent pathology, raising the striking possibility that aging is not necessarily tightly linked with costly pathologies.

However, accumulating data in both human and nonhumans suggest that not only calorie restriction but also the balance of nutrients such as protein, amino acid, fat, mineral and phytochemicals may have an important role in regulating both lifespan and health-span. Protein restriction, methionine restriction, and alternate day fasting, without overall reduction in calorie intake, are some examples of interventions with outcome similar to that observed following a calorie restricted diet regimen. There is a growing interest in this field also because, while calorie restriction may encounter limited compliance on the population scale, dietary restriction promises to have broader application. Here we review the effects of calorie restriction in different model organisms and the molecular mechanisms by which dietary interventions may modulate lifespan in simple model organisms and mammals.

## 2. CR in Yeast

The simple genetic techniques, the low cost, the possibility to do multiple tests and the short lifespan have tempted the research community to use yeast to precisely dissect the molecular mechanisms involved in nutrient responses. Glucose depletion, the most common practice to mimic calorie restriction in yeast cultures, progressively increases the mean and maximum life span when glucose concentration drops from 2% up to 0.01% [2]. On the contrary, addition of glucose to starved yeasts modifies one-third of the yeast transcriptome modulating both PKA and Sch9p activities [3]. However, Ras/PKA pathway seems to have a predominant role in this response; in fact incubation of yeast cultures with limited glucose availability (0.5%) do not further extend the lifespan of long-lived* cdc25-10* mutants (the Ras2p exchange factor) [2] or the stress resistance of* ras2* deleted mutants [4]. Phosphorylation of Bcy1p, the PKA regulatory subunit, which results in increased Bcy1p inhibitory function, seems to be involved in glucose regulation of PKA activity as well [5].

Many studies have shown that also the availability of amino acids and nitrogen bases affect the lifespan of yeasts [6]. This is consistent with the observation that mutations in genes involved in amino acid biosynthesis or nitrogen uptake influence the life span [7]. Nitrogen limitation has been linked to ROS increase and promotes autophagy induction [8, 9] by the sphingolipids biosynthetic pathway [10, 11]. The relative concentration of each available amino acid also affects yeast longevity [7, 12–15] as well as the ratio of essential to nonessential amino acids [16]. It is not surprising that single amino acid addition or depletion is sufficient to affect yeast longevity. As observed in rodents, methionine restriction extends the lifespan even of glucose-depleted cultures (0.5% glucose), while a 6-fold excess of glutamic acid has a pro-longevity effect [16, 17]. It is interesting to note that the effect of these amino acids is not influenced by* SIR2* or* SOD2* deletions whereas Gcn2p, which is a modulator of amino acid deprivation response, was shown to impair lifespan extension induced by the depletion of these amino acids [16]. Finally, since methionine restriction does not extend the lifespan of strains lacking Sch9p, the latter protein must be involved in methionine response [16]. A role for methionine in growth promotion and autophagy inhibition has been identified; this process involves the methionine product S-adenosyl-methionine that acts as a methyl donor during these processes [18]. Very recently, the role of single amino acid in the regulation of longevity pathways and stress resistance has been clarified at the molecular level [4]. The study demonstrates the existence of at least two different amino acid response pathways: the first one transduces threonine and valine through TORC1 activation; the second one transduces serine activating PDK1 orthologs Pkhsp [4, 19]. Both pathways modify Sch9p, promoting its phosphorylation at specific amino acidic residues [4]. It is interesting to note that the restriction of each of these amino acids is capable to significantly increase both the lifespan and stress resistance of yeast cultures even in the presence of high glucose concentration, thus confirming that the effect of specific amino acids is not simply due to their role as energy source.

However, the observation that extreme starvation can double maximum life span when stationary phase cells are switched into water, not only in wild type, but also in* ras2sch9* double deleted mutants cells [20], supports the hypothesis that nutrients can trigger pathways alternative to the two identified so far. Consistent with this hypothesis* RIM15* deletion, which reverses life span extension associated with the deletion of* TOR1*,* RAS2*, or* SCH9*, has only a partial effect on the life span extension under extreme CR, an observation that suggests the existence of at least another yet to be discovered prolongevity mechanism [19].

Many metabolic changes are associated with CR and some of them must be responsible for the effect on life span observed. CR accelerates ethanol and neutral lipids catabolism as well as gluconeogenesis [21, 22]. It promotes trehalose and glycogen storage, while glycogen catabolism takes place at later stages. Neutral lipids, the storage molecules free fatty acids that diacylglycerol and ergosterol are derived from, regulate energy homeostasis as well as membrane stability. In addition, they can activate apoptosis and phospholipids biosynthesis, which in turn trigger multiple transduction networks. Therefore CR, promoting lipids consumption, may have synergistic effects with many processes [16]. ATP levels are high in calorie-restricted cells; in fact, CR enhances mitochondrial activity. ROS levels are higher in cells grown on 0.2% glucose media compared to those grown on 0.5%. It has been observed that shifting the metabolism toward respiration has the same effects on lifespan and transcriptome than CR [23, 24], and that this increased respiration fuels ROS production. Therefore, probably, the amount of ROS produced with lower glucose concentration is not sufficient to damage cellular components but at the same time activates stress-protecting processes like the increase in cytosolic and mitochondrial ROS scavenging proteins (mitohormesis) [22]. It has recently been reported that such ROS production may involve epigenetic silencing of subtelomeric chromatin [25, 26].

These and other findings support the hypothesis that nutrient composition and not simply calorie restriction might be the key regulator of lifespan [12]. In particular Sch9p the appears to be the major nutrient, especially amino acids, sensing factor [4].

## 3. CR in* Caenorhabditis elegans*

The nutrition of laboratory-based nematodes relies on bacteria, mainly* E. coli*, and calorie restriction metabolic state is usually obtained either diluting these bacteria or reducing worm eating capability as well as nutrient transportation pathways. In fact, a reduction of the bacterial density by 10-fold results in 60% increased lifespan [27], whereas higher bacterial dilution can extend the lifespan of worms up to 150% [28]. Mutations in genes that affect feeding mechanics (e.g.,* eat-2*  which causes a pharyngeal pumping defect) increase life span by about 30–60% [29]. Decreased activity of the gut sodium dicarboxylate transporter NAC-3 or NAC-2 (high-affinity sodium-dicarboxylate cotransporters that accept a range of tricarboxylic acid-cycle intermediates with 4-5 carbon atoms), obtained using RNAi, produces an increase in life span varying from 15% to 19% [30, 31]. Like in other model organisms, inactivating the Ins/IGF-1 pathway significantly prolongs life span; but many experiments have shown that life span extension caused by dilution or absence of* E. coli* or eat-mutation does not completely overlap with this pathway [32, 33]. Indeed,* eat-2/daf-2* double mutant lived 20% longer than* daf-2* alone [33], and, while* daf-2* mutant lived 69% more than the wild type, the lifespan of the same mutants, grown in the absence of bacteria, increased by 274% compared to the wild type. Furthermore,* daf-2*,* daf-2/daf-12*, and* daf-16* mutants are still sensitive to nutrients as judged by SOD and catalase activities measurement [33].

Other evidences link CR to a better oxidative stress response in an insulin/IGF-1 independent way. CR response is mediated by thioredoxin 1 (trx-1) a protein that has oxidoreductase activity and is conserved in many animals. Trx-1 regulates aging and stress resistance; its deletion shortens adult lifespan and increases the sensitivity to paraquat-induced oxidative stress. It has also been discovered that* trx-1* deletion completely suppresses the lifespan increase of both the* eat-2* mutant and the dietary deprived regimen but only partially affects the lifespan of the* daf-2* mutant. At the same time* trx-1* overexpression failed to further extend the long lifespan of* eat-2* mutant. Finally,* trx-1* overexpression in the ciliated sensory neurons (ASJ) of wild-type animals extends adult lifespan but only under dietary deprivation [34].

Hansen and coworkers identified four genes extending the life span in* daf-16* (the FOXO ortholog) but not in* eat-2* mutants:* sams-1* (encoding S-adenosyl methionine synthetase),* rab-10* (encoding a Rab-like GTPase),* drr-1* (dietary restriction response, of unknown function), and* drr-2* (encoding a putative RNA-binding protein). Expression of all four genes is reduced in* eat-2* mutant suggesting these genes may be involved in longevity responses to CR [35].

More recently, Greer and Brunet proposed that specific pathways might respond to different dietary restriction regimens [36]. Low-energy sensing AMP-activated protein kinase AMPK/Aak-2 and the Forkhead transcription factor Foxo/Daf-16 are necessary for longevity induced by a CR regimen, while AMPK and Foxo are necessary for longevity induced by some but not all CR regimens.

However, the role of specific nutrients as regulators of longevity is consistent with other literature data. For example O'Rourke and coworkers have recently attributed a role to polyunsaturated fatty acids (PUFA) as regulators of longevity. The underpinning mechanism involves autophagy activation in response to PUFA supplementation [37], whereas malate and fumarate supplementation increase worm lifespan likely increasing respiration [38]. The role of amino acid supplementation has also been confirmed into this organism. Pep-2 deletion, which reduces the uptake of peptides, determines an increase in life span and stress tolerance and synergizes with reduced insulin signaling [39]. In addition, metformin, a common drug used to treat type II diabetes, increases the worm lifespan through alteration of folate and methionine metabolism [40] suggesting that amino acid metabolism may have a role different from simply being energy source also in this organism.

## 4. CR in* Drosophila melanogaster*

The idea that the effect of dietary restriction regimen on lifespan relies on the reduced intake of calories [41] was strengthened by whole-genome transcripts profile experiments in* Drosophila*. It has been observed that calorie restriction reverts the transcriptional changes normally observed during the aging process of flies and downregulates the expression of genes involved in cell growth, metabolism, and reproduction [42]. Recent experiments, however, challenged the idea that calorie restriction owes its beneficial effects on the reduced intake of calories suggesting that the depletion of specific nutrients, rather than the reduction of the overall energy intake, is responsible for the increased longevity observed in calorie restricted animals [43, 44].

A growing body of evidence points to the ratio between protein and carbohydrate (P : C), two major macronutrients, as the most important regulator of lifespan and reproduction in the fruit fly diet [45]. Higher ratio shortens lifespan whereas lower ones do the opposite [46]. A P : C ratio = 1/16 prolongs* Drosophila* lifespan, while higher protein content (P : C ratio 1/2) maximizes egg production and shortens the lifespan [47]. But it is hard to distinguish between life span extension due to protein restriction or to carbohydrate excess. Probably, both carbohydrate excess and protein depletion have crucial effects, since the longest lived flies are those which are subjected to a quite high C : P ratio and have an absolute high carbohydrate content. Because hydrolyzed yeast, the common protein source of the fruit fly, consists not only of proteins but also of vitamins, minerals, and carbohydrates, casein was used as an alternative protein source aiming at clarifying if other nutrient components could have a role in the regulation of longevity. Using this pure source of proteins overlapping results were obtained, thus confirming the major role of proteins in the aging process of this organism [48]. However, while low protein and high carbohydrate consumption maximizes lifespan a further increase of carbohydrate content does the opposite [49], probably because increasing the carbohydrate amount over a certain threshold could have other additional effects. Indeed, high carbohydrates consumption promotes obesity whereas increased protein intake suppresses adiposity, (Skorupa and coworkers). In addition, higher sucrose level enhanced the influence of proteins on lifespan, suggesting that both proteins and carbohydrates promote aging in a synergistic way [50].

One by one nutrient replenishment to otherwise severely calorie-restricted fruit fly demonstrates that only amino acids addition is effective in decreasing the lifespan and increasing fecundity, indicating that the amount of calories* per se* does not affect the lifespan [47]. In addition, essential and non-essential amino acids appear to have different roles in regulating longevity, the former being capable to negatively affect longevity, while the latter does not. The previously demonstrated tight link between longevity and fecundity is weakening since methionine addition, one of the essential amino acids, has been demonstrated to be sufficient to increase fecundity at the same extent of full feeding but had no influence on lifespan, raising the possibility that the trade-off between fecundity and longevity is not a mandatory scenario [47]. The latter observation has been confirmed by* chico*  mutants which have increased lifespan without impairment in oogenesis [51].

Other experiments suggest methionine restriction, rather than glucose depletion, as a prolongevity intervention supporting the hypothesis that the amount of macronutrients rather than the total amount of energy is the key to extend the lifespan [52]. A very recent report limits the efficacy of methionine restriction on longevity only when the overall amino acid supplementation is low thus suggesting the existence of cross talk mechanisms between the various amino acid response pathways [53].

At the molecular level, in spite of the many observations relating single diet components to life span, the underpinning molecular mechanisms have been poorly understood.

Insulin/IGF-like signaling pathway is central to control longevity in all living organisms and* Drosophila* makes no exception to this general rule. Mutations in Chico protein, the substrate of IGF1-receptor, extend fruit fly median lifespan by up to 48% in homozygotes and 36% in heterozygotes. Nevertheless some evidences suggest the existence of pathways alternative to insulin/IGF1 pathway by which nutrients can exert their action. Indeed,* chico* mutants continue to respond to CR suggesting that IIS and CR have only partially overlapping mechanisms [54]. In addition,* dFoxo* overexpression in thoracic and abdominal fat body increased longevity of 42% when flies were maintained on restricted diet, but had only a limited effect when flies were maintained on a high-yeast diet (high protein content) [55]. In addition, because null* dFoxo* mutants still respond to CR,* dFoxo*, even though its activity can modulate this response, is not the central mediator of diet response [56]. Regarding the role of ROS on lifespan, protein restricted diet, which increases lifespan in a Tor dependent manner, reduces oxidative stress resistance probably through the downregulation of antioxidant genes, while low sugar-high protein diet does the opposite; on the other hand life span increasing due to protein restriction with high sugar level is suppressed by Sod1 reduction suggesting high sugar level increases ROS production, while low protein level leads to reduced Tor signaling and promotes longevity [57].

Finally, inhibition of fatty acid synthesis or oxidation genes, in particular in the muscle tissue, inhibits lifespan extension upon DR [58].

## 5. CR in Mammals

Calorie restriction extends the lifespan of rodents [1]. This extension is accompanied with a lower incidence of most chronic diseases and results in a more youthful metabolic state [41, 59–61]. In addition, a significant proportion of the calorie-restricted rodents reaches very old age without any sign of disease [62]. CR protects from cancer [63, 64] although the underlying mechanism is not fully understood [65, 66].

One hypothesis is that energy restriction alters cell cycle regulation, inhibiting cell proliferation and increasing apoptosis [67]. On the contrary increased levels of IGF-1 reverse cancer prevention due to CR in mice probably stimulating cell proliferation and inhibiting apoptosis [68]. Notably, ames dwarf mice, which are deficient in IGF-1 production [69], postpone the incidence of neoplastic disease [70]. Other authors have reported that CR enhances the efficiency of DNA repair mechanisms therefore reducing the oxidative damage on DNA molecules [71, 72]; this is consistent with the overall upregulation of cellular and molecular defense systems during calorie restriction [73, 74].

CR attenuates aging-associated shrinkage of telomeres in many mouse tissues and reduces the incidence of tumors in mice that overexpress telomerase [75].

In male mice some of the effects of calorie restriction, such as improved physical performance, increased insulin sensitivity and reduced low-density lipoprotein as well as cholesterol levels are similar to those induced by metformin, a drug commonly used to treat type 2 diabetes. In fact, the reduction of both oxidative damage and chronic inflammation is associated with increased cellular protection [76] during metformin treatment.

The first clues that protein intake and amino acid composition could regulate mammalian longevity are derived from studies in mice and rodents. In these model systems CR causes a 40% increase in lifespan whereas protein restriction (PR) is capable of 20%, suggesting that about 50% of the CR effect on lifespan relies on PR. In addition, mtROS decreases during PR resulting in less DNA and protein oxidative damage [77].

It has been suggested that methionine restriction (MetR) could be responsible for the beneficial effects observed in protein-restricted animals [76, 78] since MetR mice have lower levels of serum IGF-1, insulin, glucose, and thyroid hormone and reduced visceral fat deposition. Levels of hormones such as leptin and adiponectin are increased in methionine-restricted animals with respect to controls and independently of overall energy restriction [79]. Furthermore, they show a delay in developing cataract and age-related changes in T-cell subclasses [80]. Conversely, methionine supplementation produces different damages on cardiovascular system [81]. Mouse has been useful also as Alzheimer's disease model. A study conducted at the Los Angeles Longevity Institute shows that periodic protein restriction cycles, without CR, in mice already displaying significant cognitive impairment and Alzheimer's disease (AD)-like pathology can promote changes in circulating growth factors (reduction of IGF-1 and increase of IGFBP-1) as well as decrease of tau phosphorylation in the hippocampus with a consequent reduction of the age-dependent impairment in cognitive performance [82].

Rats consuming no cysteine/cystine and low amount of methionine (which are the limiting amino acids for GSH synthesis) show an improvement in survival parameters and no decrease in GSH levels [83], suggesting the existence of a compensatory mechanism [84]. Likewise rats fed with 80% methionine reduction show an increase of free GSH in blood according to a drop in oxidative stress biomarkers such as plasma 8-hydoxydeoxyguanosine and 8-isoprostane, even if the activities of GSH reductase and superoxide dismutase in liver do not change [85].

Some possible mechanisms have been proposed: MetR, like PR and CR, decreases the amount of mitochondrial complex I, III, and IV in different rat tissues; excess of methionine could impair gene expression because methionine is a methyl groups donor during DNA methylation [86, 87]; furthermore, proteins rich in methionine are less resistant to oxidative modification [88, 89]; MetR avoids the production of methionine cycle metabolites like S-adenosyl-methionine, S-adenosyl-homocysteine, and homocysteine that increase the risk for degenerative diseases associated with aging and free radicals [90, 91] and represents a thiol agent that enhance mtROS production [92].

Methionine is not the only proaging amino acid in mice; in fact tryptophan has been identified as another amino acid capable of influencing the lifespan of mice and one-third restriction of this amino acid extends maximum life span by 23% [87, 93, 94].

Also lipid metabolism seems to have an important role in aging and it could be influenced by diet. Long lived mammals have tiny amount of unsaturated fatty acids in their cellular membranes, since these macromolecules are the most susceptible to oxidative stress, their depletion result in increased cellular protection against lipid peroxidation [95–99]. Sphingolipids are a class of lipids important in cellular processes for their bioactive role. Two classes of them: ceramides and glycosphingolipids are implicated in many kidney pathologies [100, 101], and sphingolipid levels change during aging in brain and liver [102]. CR prevents the accumulation of the long chain glycosphingolipids hexosylceramide and lactosylceramide (which are elevated also in fibroblasts derived from elderly humans) in the kidneys of mice during aging [103, 104]; this could be one of the mechanisms that allow CR to maintain kidney function during aging [105–111].

However, the effect of calorie restriction on primates appears to be more controversial than it has been observed in other model organisms. Two different studies on the effect of calorie restriction on rhesus monkey are presently ongoing, one at the Wisconsin National Primate Research Center (WNPRC) and another one at the National Institute of Aging (NIA). Regarding the safety of long-term calorie restriction practice both studies agree that a 30% calorie restriction, even for long term, is both feasible and safe for primates. Regarding the effectiveness of this energy-based nutritional intervention on longevity, the two studies differ since WNBRC indicates a 50% decrease in the incidence of cancer, cardiovascular diseases, type 2 diabetes, and glucose intolerance [112, 113] for the calorie restricted versus the ad libitum fed rhesus monkeys.

On the contrary, the NIA study did not find a significant improvement in survival in the calorie-restricted group. The different method used in the two studies to calculate the nutritional demands has been claimed as a possible explanation of such a discrepancy [114]. This may have led to a minor calorie reduction in the NIA study or, as very recently suggested, even the control group may have been maintained under calorie restriction diet regimen thus masking the beneficial effects of calorie restriction [115]. In addition, diet composition is quite different in the two studies; WNBRC is similar to a typical western diet whereas NIA looks more like a Mediterranean/Japanese diet thus suggesting that diet composition could underpin the different conclusions of the two studies.

However, even if there are no definitive results about the effect on human lifespan upon CR, it has been reported that this intervention protects against many age-associated pathologies in particular cardiovascular diseases like atherosclerosis and hypertension and lowers risk factors for obesity, insulin resistance, and inflammation [116, 117]. Short-term studies indicate that CR in humans lowers fasting insulin, core body temperature, and DNA damage and possibly decreases cancers [62, 118].

It has also been demonstrated that humans with growth hormone receptor deficiency also exhibit a high reduction of IGF-1 and insulin level resulting in a highly reduced incidence of cancer and diabetes mortality [119]. Another study has reported a similar protection from cancer development in GHRD [120]. On the other hand, protein restriction or the depletion of specific amino acid, namely, methionine and tryptophan, from the diet has the potential to reduce the level of the circulating IGF-1 and to increase the level of the IGF-1 binding proteins [121–123]. Consequently, similar dietary regimen inhibits tumor growth in human xenograft models [124]. It is interesting to note that the observed association between protein restriction and lower free IGF-1 is independent from calorie intake and relies only on diet composition. Recent epidemiological and cellular studies have confirmed the association between protein consumption and IGF-1 level in humans [125]. In addition, the group consuming a high protein diet has a fourfold risk developing a cancer and a 75% enhanced risk of all causes of death. It must also be noted that the detrimental effect of the high protein diet on 65 and younger is counterbalanced by a milder positive effect on older people raising the possibility that aging should be considered as a dynamic process and that each phase of this process has different nutritional demands.

## 6. Conclusions

The usefulness of calorie restriction diet regimen has been demonstrated in all the species tested from the simplest unicellular eukaryotes to mammals. Even the discrepancies between the two primate studies have recently been solved confirming the efficacy of calorie restriction also in these long-lived species. However, recent research articles suggest that the effect of calorie restriction relies on the reduced uptake of single component of the diet and not on the overall energy uptake. Protein restriction and variations in the ratio between macronutrients demonstrated their efficacy in several model systems including humans. Methionine restriction efficacy has been confirmed in most species although the molecular mechanism is not yet fully understood.

In addition, the molecular mechanism underlying the effect of selected amino acids has recently been clarified in simple model organisms suggesting their role as longevity regulators. Human studies have also revealed that nutritional intervention may have different outcomes at different ages suggesting caution transferring the results obtained in model systems to human.
